# Supplementary material for: Insights into Repeated Renal Injury Using RNA-Seq with Two New RPTEC Cell Lines
Source: Int J Mol Sci. 2023 Sep 18;24(18):14228. doi: 10.3390/ijms241814228 (PMC10531624; doi:10.3390/ijms241814228)
Supplement: Supplementary file 1 [file ijms-24-14228-s001.zip › Figure S1.pdf]

Experiment 1: Concentration-Response to CisPt or AFB1 at 72 hr

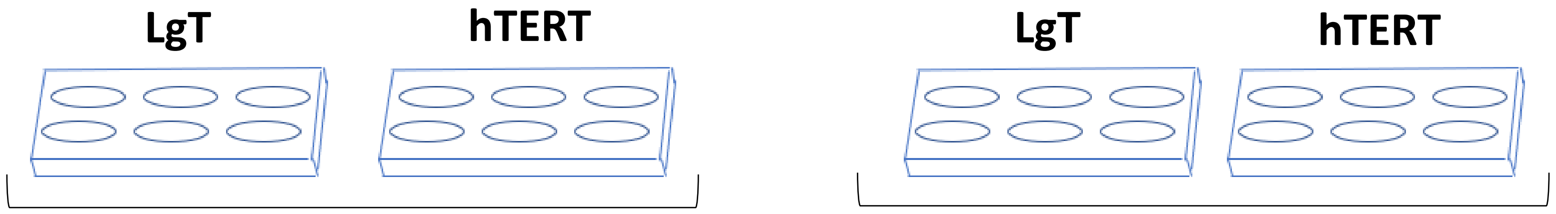

AFB1: 0.0 μM
12.5 μM
25 μM
50 μM
75 μM
100 μM

CisPt: 0.0 μM
0.25 μM
1.00 μM
1.50 μM
2.00 μM
2.50 μM

Experiment 2: Time Course-Response to 2.5 μM CisPt

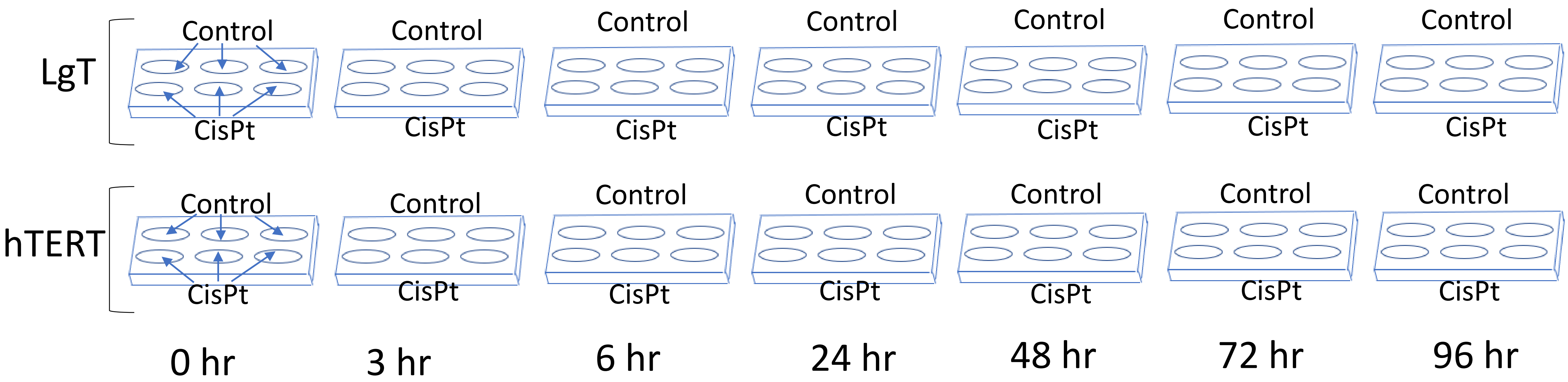

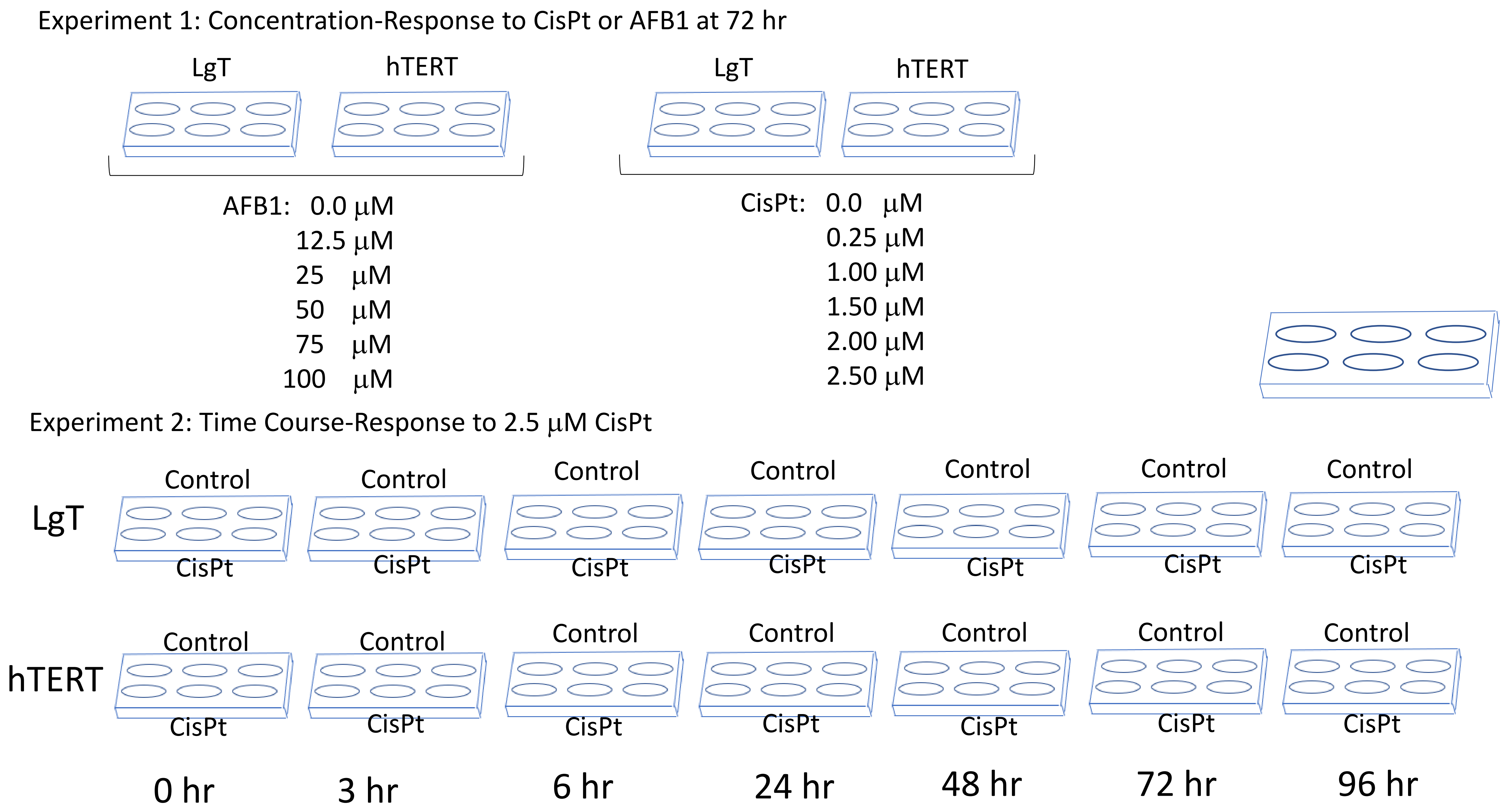
Experiment 1: Concentration-Response to CisPt or AFB1 at 72 hr
LgT
hTERT
LgT
hTERT
AFB1: 0.0 μM
12.5 μM
25 μM
50 μM
75 μM
100 μM
CisPt: 0.0 μM
0.25 μM
1.00 μM
1.50 μM
2.00 μM
2.50 μM
Experiment 2: Time Course-Response to 2.5 μM CisPt
LgT
hTERT
Control
CisPt
0 hr
3 hr
6 hr
24 hr
48 hr
72 hr
96 hr
