# Supplementary material for: Insights into Repeated Renal Injury Using RNA-Seq with Two New RPTEC Cell Lines
Source: Int J Mol Sci. 2023 Sep 18;24(18):14228. doi: 10.3390/ijms241814228 (PMC10531624; doi:10.3390/ijms241814228)
Supplement: Supplementary file 1 [file ijms-24-14228-s001.zip › Table S9.pdf]

Authentication of LgT and hTERT moRPTEC cell lines as mouse.

Sample Designation: LgT moRPTEC[ǂ]

% Match to Mouse	100%
Locus	Query Profile*
18-3	17,19,21,22
4-2	19.3, 21.3
6-7	15
19-2	12
1-2	13
7-1	29
1-1	8,11
3-2	12
8-1	15
2-1	9
15-3	21.3, 23.3
6-4	17.2, 18, 18.2
11-2	18
17-2	13,14,16
12-1	19
5-5	12,16
X-1	26
13-1	15, 15.2

*Total #alleles in query sample profile = 30

Sample Designation: hTERT moRPTEC[ǂ]

% Match to Mouse	100%
Locus	Query Profile*
18-3	17,21
4-2	19.3
6-7	15
19-2	12
1=2	13
7-1	29
1-1	8,10
3-2	10,12
8-1	15,16
2-1	9
15-3	21.3, 23.3
6-4	18.2
11-2	14,18
17-2	13,14
12-1	19
5-5	12,14
X-1	26
13-1	15

*Total #alleles in query sample profile = 26

[ǂ] The ATCC (Manassas, VA), Mouse Cell Authentication Service (Cat No. 137-XV) was used for moRPTEC LgT and hTERT cell lines. Testing involves analysis for presence of 18 Mouse short tandem repeat (STR) loci accompanied by 2 additional markers (Human D8 and D4) to screen for the presence of human or African green monkey species. Each sample was processed using the ABI Prism® 3500xl Genetic Analyzer. Data were analyzed using GeneMapper® ID-X v1.2 software (Applied Biosystems). Appropriate positive and negative controls were run and confirmed for each assay set. The LgT and hTERT moRPTEC cell lines were both authenticated as mouse cell lines.
